# Synovial Lipomatosis of the Knee: A Case Report and Review of the Literature

**DOI:** 10.7759/cureus.99587

**Published:** 2025-12-18

**Authors:** El Kacem El Marbouh, Abderrahim Majjad, Said Akjouj, Mohammed Boussaidane, Abderrahim El Ktaibi, Oumaima Mekhakh, Hamza Toufik, Leila Taoubane, Ahmed Bezza

**Affiliations:** 1 Rheumatology, Mohammed V Military Training Hospital, Rabat, MAR; 2 Radiology, Agdal Clinic, Rabat, MAR; 3 Orthopaedics, Mohammed V Military Training Hospital, Rabat, MAR; 4 Pathology, Mohammed V Military Training Hospital, Rabat, MAR

**Keywords:** arborescent lipoma, chronic monoarthritis, knee joint, synovectomy, synovial lipomatosis

## Abstract

Synovial lipomatosis (lipoma arborescens) is a rare, benign, intra-articular lesion characterized by villous proliferation of mature adipocytes, most commonly affecting the knee. Early recognition is essential to prevent misdiagnosis and long-term joint dysfunction. MRI is highly suggestive, while histopathology confirms the diagnosis. We describe the case of a 34-year-old male with persistent left knee monoarthritis. Laboratory investigations, including complete blood count, erythrocyte sedimentation rate, and C-reactive protein, were within normal limits. MRI showed frond-like synovial projections with fat-signal intensity suppressed on fat-saturated sequences. Histopathology confirmed the diagnosis of synovial lipomatosis. The patient underwent synovectomy, resulting in full resolution of symptoms and no recurrence during four months of follow-up. Synovial lipomatosis should be considered in patients with chronic knee effusion. MRI is usually diagnostic, and early arthroscopic synovectomy restores function, prevents degenerative changes, and achieves excellent outcomes.

## Introduction

Synovial lipomatosis (lipoma arborescens) is a rare articular lesion consisting of subsynovial villous proliferation of mature fat cells [1]. The disease is generally identified in the knee joints, with a lower predilection for other joints [2], such as the elbows, shoulders, and wrists [3]. The reported prevalence is low, with some estimates suggesting it accounts for approximately 0.3-0.7% of intra-articular lipomatous lesions, though precise epidemiological data remain limited [4]. Patients typically present with chronic joint swelling due to recurrent effusion, sometimes accompanied by mechanical discomfort.

MRI plays a central role in diagnosis by demonstrating villous synovial proliferation with characteristic fat-signal intensity [5]. Histological confirmation, however, remains the gold standard. Management relies mainly on surgical synovectomy, open or arthroscopic, which effectively removes the proliferative fatty synovium and prevents recurrence [1]. Early recognition of this uncommon entity is essential to avoid misdiagnosis and reduce the risk of long-term joint dysfunction. Here, we report a case of synovial lipomatosis of the knee in a 34-year-old man, followed by a concise literature review.

This article was previously presented as a meeting abstract at the 35th National Congress of the Moroccan Society of Rheumatology on May 29-31, 2025.

## Case presentation

A 34-year-old man with no significant past medical history presented with a five-month history of progressive swelling of the left knee. The swelling was initially painless but gradually became spontaneously painful, leading to functional discomfort. He reported no history of trauma, repetitive stress, previous similar episodes, obesity, or metabolic disorders.

**Figure 1:**
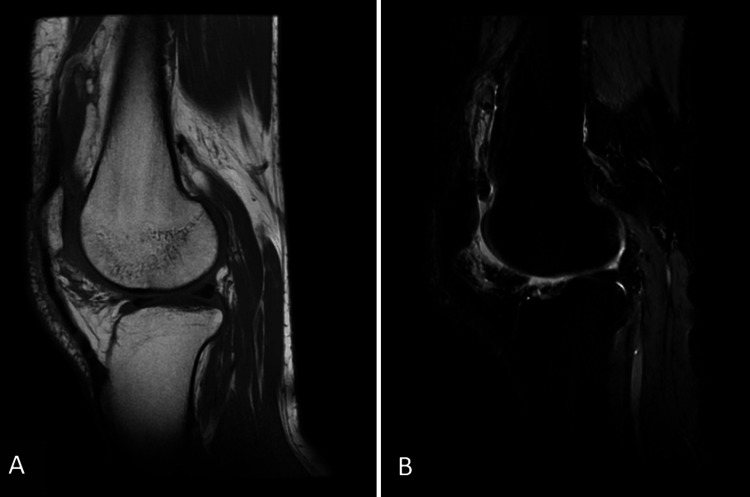
Sagittal MRI of the knee. (A) T1-weighted image demonstrating frond-like synovial proliferation with high signal intensity identical to subcutaneous fat. (B) Sagittal proton density fat-suppressed image showing complete signal suppression of the synovial lesions, confirming their fatty nature.

Musculoskeletal assessment revealed a slightly impaired but independent gait. The left knee was swollen without signs of local inflammation. The patellar tap test was positive, while joint mobility remained preserved. Abdominal, pulmonary, and neurological examinations were unremarkable. Hematological and inflammatory parameters, including complete blood count, erythrocyte sedimentation rate, and C-reactive protein, were all within normal limits. Sputum cultures for tuberculosis were negative. Synovial fluid analysis from the knee aspiration demonstrated a non-inflammatory profile, characterized by a viscous, yellowish appearance, with a white blood cell count of 160/mm³ (<200/mm³) with 34% polymorphonuclear leukocytes. Glucose and protein levels were not measured. Bacterial cultures were negative. The knee X-ray was normal. MRI of the knee revealed frond-like synovial proliferation within the joint. The lesions demonstrated high signal intensity on sagittal T1-weighted images, similar to subcutaneous fat. On sagittal proton density fat-suppressed sequences, complete signal suppression of the synovial proliferation was observed, confirming the fatty nature of the lesions. These imaging findings were characteristic of synovial lipomatosis (Figure 1).

**Figure 2:**
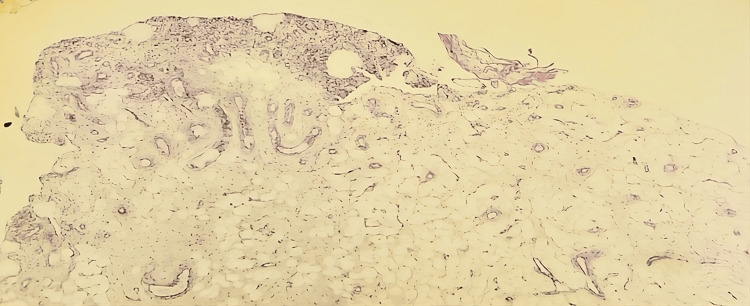
Microscopic examination showing benign synovial tissue with subepithelial infiltration by mature adipocytes.

An ultrasound-guided synovial biopsy demonstrated chronic nonspecific synovitis. A surgical biopsy with total synovectomy was performed. Histopathological examination (hematoxylin and eosin stain, ×20 magnification) demonstrated villous proliferation of mature adipocytes beneath an enlarged reactive synovial lining, associated with mild chronic inflammatory infiltrates (Figure 2).

The postoperative course was uneventful, with a tailored functional rehabilitation program. No tumor recurrence was observed after four months of follow-up.

## Discussion

Synovial lipomatosis is a rare benign lesion of the synovium, most often involving the suprapatellar pouch of the knee [2]. This condition is rare, and its exact prevalence remains uncertain due to potential underdiagnosis or asymptomatic presentations. It predominantly affects adults [6] and typically presents in the fourth to sixth decades of life, though cases in younger individuals have been reported [7]. Our patient, a 34-year-old man with no identifiable risk factors, reflects the typical presentation of this condition in otherwise healthy adults.

Several case series and reports have described lipoma arborescens of the knee. Vilanova et al. reported 33 cases, all showing frond-like fatty projections on MRI [8]. Howe and Wenger documented 45 lesions, distinguishing typical knee involvement from atypical or polyarticular forms [9]. Our patient aligns with the typical pattern: monoarticular knee involvement with characteristic MRI and histopathological findings, and no systemic disease.

Although the exact cause of synovial lipomatosis remains unclear, it has been linked to systemic conditions such as fat metabolism disorders (e.g., short bowel syndrome) [10]. It has also been associated with trauma, inflammation, and developmental and neoplastic processes [6]. At the molecular level, studies have identified a potential role for signaling pathways involved in adipogenesis, such as the peroxisome proliferator-activated receptor-gamma (PPAR-γ) pathway [11]. PPAR-γ is a key regulator of preadipocyte differentiation into mature adipocytes, and its excessive activation may contribute to the proliferation of synovial adipose tissue.

Clinically, patients usually present with painless swelling due to chronic effusion, sometimes associated with activity-related pain [12]. Pain can range from mild discomfort to severe, especially during activity.

MRI helps in early diagnosis by defining its nature and anatomical extensions. Characteristic features include villous or nodular projections with high T1/T2 signal intensity that suppresses on fat-saturation sequences, while non-fatty synovium demonstrates heterogeneous signal intensity [12].

Histology confirms the diagnosis. Macroscopically, the lesion appears frond-like with yellow fatty villi, and microscopically, it consists of mature adipose tissue, often with congested capillaries and mild chronic inflammation, and the synovial cells may appear to be enlarged and reactive, with abundant eosinophilic cytoplasm [13].

Differential diagnosis of synovial lipomatosis encompasses synovial chondromatosis, pigmented villonodular synovitis (PVNS), and rheumatoid arthritis, each exhibiting distinct imaging characteristics [6]. Synovial chondromatosis shows calcified cartilaginous nodules [14]. PVNS is characterized by hemosiderin-laden synovial masses with T2 hypointensity. Rheumatoid arthritis presents with synovial hypertrophy and erosions, unlike the fatty infiltration of synovial lipomatosis [13] (Table 1).

**Table 1:** Key conditions mimicking synovial lipomatosis in chronic knee effusion.

Condition	Clinical features	MRI findings	Histology
Lipoma arborescens	Chronic painless effusion	Frond-like fatty projections, fat-sat suppression	Mature adipocytes
Pigmented villonodular synovitis	Swelling, pain	Low T2 signal, hemosiderin	Fibroblasts, hemosiderin-laden macrophages
Synovial chondromatosis	Swelling, locking	Nodular/cartilaginous, possible calcification	Cartilaginous nodules
Rheumatoid arthritis (rare monoarthritis)	Pain, stiffness, systemic signs	Synovial hypertrophy, joint erosions	Synovial inflammation, pannus

Surgical synovectomy remains the gold standard for the treatment of symptomatic synovial lipomatosis. Arthroscopic techniques offer a minimally invasive option with quicker recovery compared to open procedures [15]. Recurrence is rare, though some patients may develop recurrent effusions [1]. In our case, the patient showed no signs of recurrence during the four-month follow-up period.

## Conclusions

Synovial lipomatosis is a rare but clinically important cause of chronic knee effusion, predominantly affecting the suprapatellar pouch. MRI is usually diagnostic, demonstrating characteristic frond-like fatty synovial projections with suppression on fat-saturated sequences. Histopathology confirms the diagnosis. Early surgical intervention, particularly arthroscopic synovectomy, restores joint function and prevents long-term degenerative changes. Recurrence after complete excision is uncommon. Clinicians should consider this entity in patients with unexplained chronic knee swelling to ensure timely diagnosis and management.
